# Thermally Conductive and Antistatic Properties of Silicone Rubber Reinforced by the Modified Graphene Oxide

**DOI:** 10.3390/polym14214703

**Published:** 2022-11-03

**Authors:** Deling Li, Liming Dong, Ying Chen, Congcong Luo, Jun Zhou, Guangtian Liu, Haidong Ren

**Affiliations:** 1 College of Materials and Chemical Engineering, Xuzhou University of Technology, Xuzhou 221018, China; 2 School of Environment and Chemical Engineering, Yanshan University, Qinhuangdao 066004, China; 3 State Key Laboratory of Advanced Materials and Electronic Components, Guangdong Fenghua Advanced Technology Holding Co., Ltd., Zhaoqing 526020, China

**Keywords:** silicon rubber, graphene oxide, antistatic property, thermal conductivity, nanocomposite

## Abstract

Silicone rubber (SR)/vinyl-graphene oxide (vinyl-GO) nanocomposites were prepared through the hydrosilylation reaction of silicon hydrogen polydimethylsiloxane (H-PDMS) with vinyl polydimethylsiloxane (vinyl-PDMS), in which vinyl-GO was used as a nano filler. The thermally conductive and antistatic properties of the nanocomposites, and their tensile strength and thermal stability were evaluated. The thermally conductive and antistatic properties increased naturally when the nanocomposites had eight to nine parts of vinyl-GO. The addition of 9 parts of vinyl-GO increased the thermal conductivity to 0.44 from 0.17 W/m^−1^·K^−1^ of neat SR and the surface resistance value to 10^8^ from 10^14^ Ω of neat SR. Vinyl-GO is effective in improving the tensile strength and toughness of the nanocomposites. The tensile strength and elongation at break of the nanocomposites were much higher than that of neat SR, especially for 10 parts of vinyl-GO in the nanocomposite, and the tensile strength was 1.84 MPa and the elongation at break was 314.1%. Additionally, compared with neat SR, the nanocomposites had a much higher thermal stability. For eight parts of vinyl-GO in the nanocomposites, H-PDMS with the selected silicon hydrogen content and vinyl-PDMS with the selected vinyl content could offer an appropriate cross-linking degree that suits the character of GO. When the nanocomposite had eight parts of vinyl-GO, its scanning electron microscope exhibited a monolayer GO with folded, twisted, and local surface folds. However, there was a certain amount of multilayer aggregation of GO for 10 parts of vinyl-GO in the nanocomposite.

## 1. Introduction

The integrity of electronic components offered by electronic potting adhesive has the characteristics of water repellency, insulation, and resistance to the external impact and vibration [1,2,3,4,5,6]. Epoxy resin is commonly used as an electronic potting adhesive. However, it is prone to brittle fracture after curing due to the rigidity of the cross-linked chain. Thus, a component or line peels off integral electronic components under external forces [7,8,9,10,11]. SR exhibits excellent toughness, high and low temperature resistance, weather resistance, and thermal oxygen stability because of its backbone of Si-O [12,13,14,15]. Compared with epoxy resin, SR plays a much more satisfactory role in eliminating internal stress and protecting the integrity of electronic components in a wide temperature range due to its flexible cross-linked chain [16,17,18]. Additionally, the electronic potting adhesive of SR has an outstanding resistance to yellowing in long-term service [19,20]. However, the thermally conductive and antistatic properties of SR are poor, and the heat generated from the electronic components cannot dissipate in time. Thus, the heat and accumulated charge on the surface of SR limit its extensive application in electronic potting for precision electronics and electronic components [20,21,22,23]. It is necessary to obtain SR with progressive thermal conductivity and antistatic properties.

The incorporation of nano fillers with polymers is an effective means for the fabrication of thermally conductive and antistatic polymer composites [18,20,21,22,23]. Monolayer graphene has a high carrier mobility of 200,000 cm^2^/(V·s) [24] and a high thermal conductivity of 5300 W/m^−1^·K^−1^ [25,26]. Graphene and its derivatives have been considered as nano fillers to improve the thermally conductive and antistatic properties of polymer matrix due to their perfect characteristics [27,28,29,30]. However, one common issue is the poor interfacial compatibility of graphene or its derivatives with the polymer matrix due to its two-dimensional morphology with a high aspect ratio. This poor interfacial compatibility leads to serious aggregation and poor enhancement effects.

Improved interfacial compatibility could allow polymer-based nanocomposites to achieve satisfactory thermal conductivity or antistatic properties [31,32,33,34,35,36]. Hu Chen et al. [31] reported that graphene modified by 3-glycidyloxypropyltrimethoxysilane allows uniform dispersion in polymers. Lin Yong et al. [32,33] successfully prepared graphene modified by zinc methacrylate and found that with the addition of 40 parts of the modified graphene, the resistivity of the natural rubber decreased by 3 orders of magnitude.

SR reinforced with graphene or its derivatives through the vulcanization reaction of SR with peroxide has been reported [35,36]. The cross-linked structures in the reported SR nanocomposites are formed from radical initiation. It is difficult to control the whole or local cross-linking degree of the SR matrix due to the random characteristics of the radical reaction. For the cross-linked SR, its cross-linked structures are a critical factor in obtaining a preferable dispersion of GO in SR due to the large specific area and two-dimensional geometry of GO. Consequently, a preferable dispersion of GO in SR would lead to a preferable SR nanocomposite. Thus, the preferable dispersion of graphene or its derivatives in the SR matrix and the resulting properties of SR nanocomposites are still a severe challenge. In this paper, SR/vinyl-GO nanocomposites with improved thermally conductive and antistatic properties were prepared via in situ hydrosilylation reaction of H-PDMS with vinyl-PDMS in the presence of vinyl-GO. The cross-linking degree of the SR matrix is controllable because of the given structures of H-PDMS and vinyl-PDMS. Here, GO was modified by vinyltriethoxysilane to maintain a suitable dispersion of GO during the hydrosilylation reaction. In this work, the tensile strength, thermal stability, thermal conductivity, and antistatic properties of the nanocomposites were estimated. The dispersion of vinyl-GO in the SR matrix was observed using field emission scanning electron microscopy.

## 2. Materials and Methods

### 2.1. Materials

H-PDMS, vinyl-PDMS, and Karstedt’s catalyst solution in dimethylbenzene (2%) were purchased from Shanghai Guiyou New Materials Co., Ltd. (Shanghai, China). H-PDMS is a structure that contains terminal silicon hydrogen and side silicon hydrogen, and its silicon hydrogen content is 10.0, 5.0, 3.0, and 1.8 mmol·g^−1^, respectively. Vinyl-PDMS is a vinyl-terminated linear structure, and its vinyl content is 1.3, 1.1, 0.9, 0.7, 0.4, and 0.2 mmol·g^−1^, respectively. GO with a diameter of 0.5–5 μm and a thickness of 0.8–1.2 nm was purchased from Beijing Dekedaojin Technology Co., Ltd. (Beijing, China). Vinyltriethoxysilane and anhydrous alcohol were purchased from Tianjin Fuyu Fine Chemical Co., Ltd. (Tianjin, China).

### 2.2. Preparation of the Modified GO

In total, 30.0 g GO, 8.0 g vinyltriethoxysilane, 1 mL H_2_O, and 300 mL anhydrous alcohol were ultrasonically mixed in a 3-mouth flask at 0 °C until GO was uniformly dispersed in anhydrous alcohol. The mixture was heated to 78 °C, and the reaction was continued for 2 h. The mixture was separated by centrifugation at 8000 r·min^−1^, and the solids obtained were the crude vinyl-GO. The crude vinyl-GO was further dispersed in anhydrous alcohol, ultrasonicated for 30 min at 0 °C, and centrifuged at 8000 r·min^−1^. The collected solid was washed with anhydrous alcohol. To obtain vinyl-GO free from vinyltriethoxysilane, the cycle of dispersion–centrifugation in anhydrous alcohol was conducted several times until no residue was detected from the evaporation of the supernatant solution.

### 2.3. Preparation of Organosilicon Compound

This preparation procedure was conducted according to [37]. According to the silicon hydrogen content of 10.0, 5.0, 3.0, and 1.8 mmol·g^−1^, the mass of H-PDMS was 32.4, 16.2, 16.4, and 9.0 g, respectively. The mixture of H-PDMS was stirred vigorously in a jacketed beaker for 30 min at 25 °C. The total content of silicon hydrogen in this mixture was 470.4 mmol. According to the vinyl content of 1.3, 1.1, 0.9, 0.7, 0.4, and 0.2 mmol·g^−1^, the mass of vinyl-PDMS was 60, 200, 60, 40, 20, and 20 g, respectively. The mixture of vinyl-PDMS was vigorously stirred in another jacketed beaker for 2 h min at 25 °C and ultrasonically dispersed for 30 min at 25 °C. The total content of vinyl in this mixture was 392.0 mmol. The molar ratio of silicon hydrogen with vinyl was 1.2. The two mixtures were mixed by vigorous stirring, simultaneously accompanied with ultrasonic dispersion for 2 h at 25 °C.

### 2.4. Curing of Organosilicon Compound

In total, 50 μL Karstedt’s catalyst solution was mixed into 40 g organosilicon compound by vigorous stirring for 5 min at 25 °C, simultaneously accompanied with ultrasonic dispersion. The mixture was immediately poured into a dumbbell mold and completely defoamed in a vacuum. The organosilicon compound was pre-cured at 110 °C for 2 h, cured at 130 °C for 4 h, and post-cured at 160 °C for 2 h. Sheets of neat SR were obtained.

### 2.5. Curing of SR/Vinyl-GO Nanocomposites

A certain amount of vinyl-GO was mixed into 40 g organosilicon compound by vigorous stirring for 2 h at 25 °C, simultaneously accompanied with ultrasonic dispersion. Then, 50 μL of Karstedt’s catalyst solution was added and continuously mixed for 5 min. The mixture was immediately poured into a dumbbell mold and completely defoamed in a vacuum. The organosilicon compound was pre-cured at 110 °C for 2 h, cured at 130 °C for 4 h, and post-cured at 160 °C for 2 h. Sheets of SR/vinyl-GO nanocomposites were obtained.

### 2.6. Characterization

A certain amount of vinyl-GO was ultrasonically dispersed in anhydrous alcohol until a uniform suspension was obtained. The suspension flowed naturally into a clean and dried glass plate. Alcohol was volatilized at room temperature and then evaporated in a vacuum at 50 °C. The resulting thin film on the glass plate was the vinyl-GO film. The contact angle of the vinyl-GO film was determined by a contact angle measuring instrument (JC2000DM) at 25 °C. The same procedure was performed for the estimation of the contact angle of GO. 

The FTIR spectra of the GO film and the vinyl-GO film were recorded on an FTIR spectrometer (Bruker Vertex 70) via the ATR method.

The tensile strength and elongation at break of SR and SR/vinyl-GO nanocomposites were estimated by the electronic universal tensile tester (KT877S) with a test force of 2 kN and a tensile rate of 5 mm·min^−1^. Five parallel sheets for every component were tested, and the average value was calculated. 

The thermal stability of GO, vinyl-GO, SR, and SR/vinyl-GO nanocomposites is determined by thermal gravimetric analysis (TGA 4000, PE). About 6 mg samples are heated from 30 to 800 °C at a heating rate of 10 °C·min^−1^. N_2_ flow rate is kept at 20 mL·min^−1^.

The thermal conductivity of the SR and SR/vinyl-GO nanocomposites was determined by a thermal conductivity tester (heat flow method, DRL-III) at 25 °C. Five parallel sheets were tested for each component, and the average value was calculated. 

The volume resistivity of the SR and SR/vinyl-GO nanocomposites was determined by a surface resistance tester at 25 °C. 

The dispersion of vinyl-GO in the SR matrix was analyzed using field emission scanning electron microscopy (SEM, Sigma300, Zeiss, Germany). The tested samples were obtained from the fresh tensile fracture sheets of the SR/vinyl-GO nanocomposites.

## 3. Results

### 3.1. Characterization of Vinyl-GO and GO

**Figure 1:**
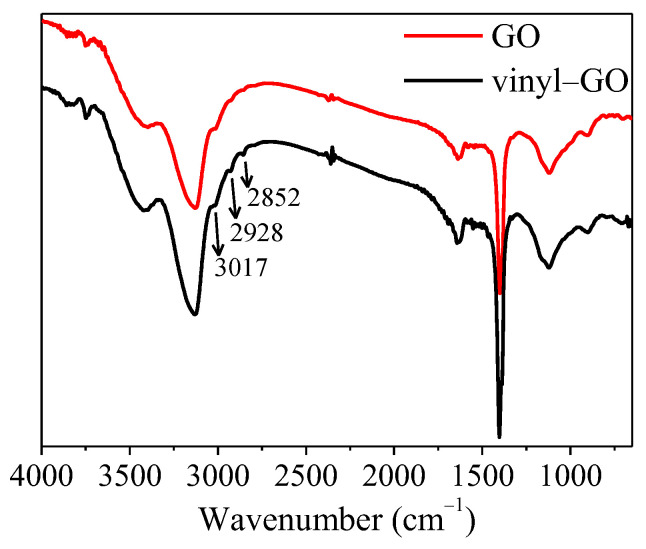
FTIR spectra of GO and vinyl−GO.

The FTIR spectra of GO and vinyl-GO are shown in Figure 1. Compared with that of GO, the peaks at 3017, 2928, and 2852 cm^−1^ appearing in the spectra of vinyl-GO suggest that an alkyl or double bond exists on the surface of vinyl-GO. It can be inferred that vinyltriethoxysilane might be covalently bonded to the surface of GO to a certain extent.

**Figure 2:**
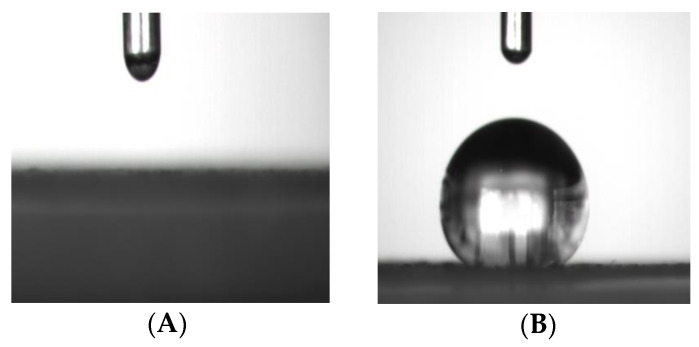
The graphs of water droplets after dripping on the surface of GO and vinyl-GO for 5 s. (**A**) The surface of GO; (**B**) the surface of vinyl−GO.

This presumable grafting of vinyltriethoxysilane onto GO could be further clarified by comparing the contact angle between GO and vinyl-GO. Figure 2A shows the graph of a water droplet that dripped on the surface of GO for 5 s. This indicates that GO exhibits good hydrophilicity due to the existence of a large number of hydroxyl and carboxyl groups on its surface. The hydrophilicity of GO accounts for the poor interfacial compatibility of GO with the SR matrix. It is necessary for GO to adjust its hydrophilic surface into a hydrophobic surface. The silane coupling agent is frequently considered as a suitable functional molecule for GO because of its high content of hydroxyl groups [22,31,35]. Considering the two-dimensional morphology of GO in this work, vinyltriethoxysilane was selected because of its short chain in this work. The contact angle of vinyl-GO is 133° (Figure 2B), suggesting that vinyl-GO has a hydrophobic surface and presumably has a good interfacial compatibility with SR. Generally, a good interfacial compatibility in organic–inorganic composites accounts for a high reinforced thermal conductivity, thermal stability, and other properties [31,32,33,34,35,36].

**Figure 3:**
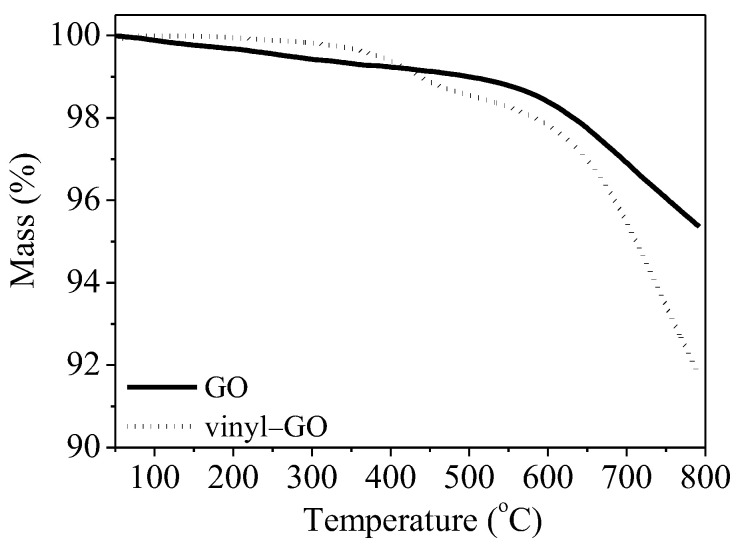
TG curves of GO and vinyl−GO.

The thermal stability of GO and vinyl-GO also provides a hint of the covalent bond formed from the reaction of vinyltriethoxysilane with GO. As shown in Figure 3, the mass loss of GO is obviously higher than that of vinyl-GO at a temperature below 400 °C, indicating that vinyl-GO has a low content of adsorbed water and oxygen-containing groups on its surface. As previously observed, vinyl-GO has a hydrophobic surface. The mass loss of GO within the range of 150–240 °C is reported to be caused by the pyrolysis of functional groups on its surface [35]. The mass loss of vinyl-GO is probably due to the decomposition of unreacted oxygen-containing functional groups and vinyltriethoxysilane grafted onto GO. It is appropriate to speculate that the oxygen-containing functional groups on the GO surface partially react with the vinyltriethoxysilane. Consequently, the total mass loss of vinyl-GO is much higher than that of GO.

### 3.2. Tensile Properties of SR/Vinyl-GO Nanocomposites

**Scheme 1:**
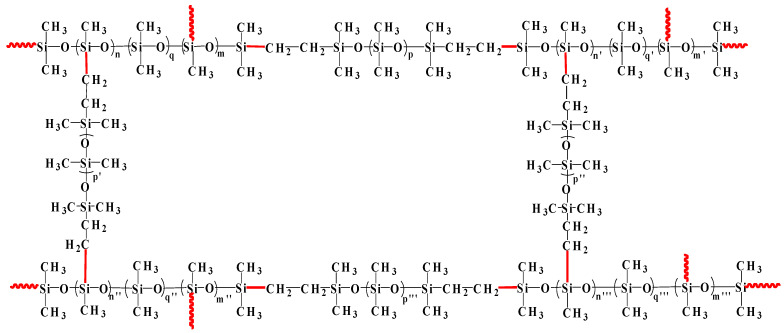
The schematic cross-linking structure of SR. The red solid lines represent the bonds originating from the hydrosilylation reaction of vinyl with silicon hydrogen, and the wavy lines represent the chains of linear PDMS.

The hydrosilylation reaction of vinyl of vinyl-PDMS and silicon hydrogen of H-PDMS proceeded under the catalysis of Karstedt’s catalyst. H-PDMS with various silicon hydrogen contents and vinyl-PDMS with various vinyl contents were selected based on the character of GO and evaluated in our previous work [37]. H-PDMS, serving as a cross linker, consists of several H-PDMS with varied silicon hydrogen contents and plays a role in adjusting the local cross-linking structures. The higher the silicon hydrogen content of H-PDMS, the higher the side silicon hydrogen content. Basically, the local cross-linking degree might be increased while vinyl-PDMS consists of several vinyl-PDMS with varied vinyl contents and determines the chain length of SR. Vinyl-PDMS with a lower vinyl content leads to a longer chain length of SR. In order to actualize the utmost reaction degree of vinyl-PDMS, the molar ratio of silicon hydrogen to vinyl was 1.2:1. In this molar ratio, the suspended chains in the cross-linking structure of SR are avoided to the most extent. Consequently, SR has a better tensile strength. The schematic cross-linking structure of SR is shown in Scheme 1. 

**Figure 4:**
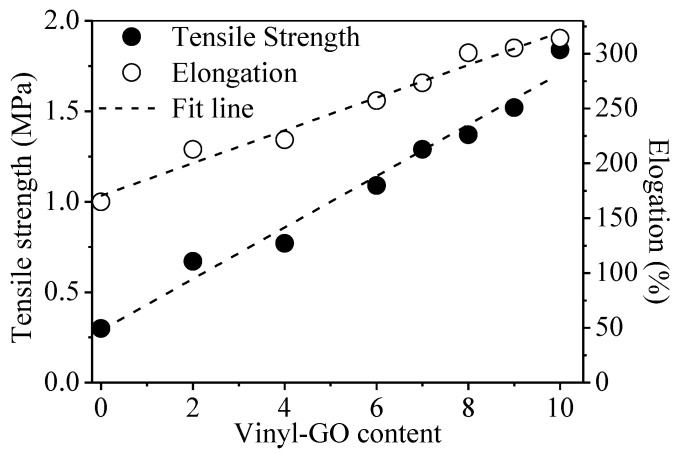
The tensile properties of SR/vinyl-GO nanocomposites.

The tensile strength of neat SR is 0.3 MPa, and its elongation at break is 165.0% while the tensile strength and elongation at break of the SR/vinyl-GO nanocomposites are much higher than that of neat SR and increase with the increasing vinyl-GO content, as shown in Figure 4. Especially for 10 parts of vinyl-GO, the tensile strength is 1.84 MPa and the elongation at break is 314.1%. In addition, the linear growth of the tensile properties with the increasing vinyl-GO content gives a hint of the uniform dispersion of vinyl-GO in the SR matrix. These results indicate that vinyl-GO has an appreciably enhanced effect on the tensile properties of the nanocomposites. Vinyl-GO could act as a stress center to efficiently absorb or transfer the energy of external action, thus increasing the tensile strength of SR. It has been reported that uniformly distributed nano fillers can dissipate external stress by an efficient stress transmission [29,30,31,32,33].

The magnitude for the elongation at break is unremarkable, varying from 165.0% to 314.1%. There might be two presumable reasons for this phenomenon. One is that vinyl-GO embedded in the cross-linked network retards the chain mobility due to its two-dimensional geometry. The other is that the vinyl on the surface of vinyl-GO might react with the silicon hydrogen of H-PDMS, thereby promoting the local cross-linking degree. The increased local cross-linking degree also limits the chain mobility of SR. 

### 3.3. Thermal Stability of SR/Vinyl-GO Nanocomposites

**Figure 5:**
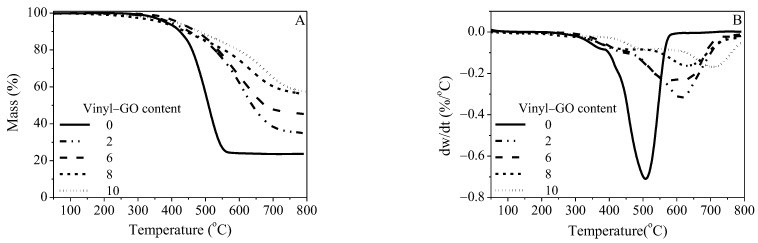
TG curves (**A**) and DTG curves (**B**) of neat SR and SR/vinyl-GO nanocomposites.

The TG and DTG curves of the neat SR and SR/vinyl-GO nanocomposites are shown in Figure 5. The residue ratios of the completely degraded SR/vinyl-GO nanocomposites are much higher than that of the neat SR, as shown in Figure 5A. There are two major peaks on their DTG curves, ascribed to two thermal degradation stages, suggesting that neat SR and SR/vinyl-GO nanocomposites have similar thermal degradation mechanisms. The two major peaks of the maximum weight loss rate are identified as *T_max,1_* and *T_max,2_*, respectively. Compared with that of neat SR, the two degradation stages of the SR/vinyl-GO nanocomposites obviously shift to higher regions. Thus, vinyl-GO could obviously improve the thermal stability of SR but had no effect on the degradation process of SR.

The reported depolymerization mechanisms of linear polydimethylsiloxane (PDMS) involve two competing processes [38,39]. One is the process of the intramolecular cyclic transition state and the scission of cyclic oligomers within a chain until the linear structure is too short to cyclize. The other is a radical mechanism that is speculated to occur due the formation of methane originating from the scission of the Si-CH_3_ bond at a higher temperature. However, for the cross-linked PDMS, obtained from the reaction of vinyl and Si-H catalyzed by Karstedt’s catalyst, the cyclic transition state is seriously restricted due to the cross-linking structures and the detained Karstedt’s catalyst. Due to the intervention of Karstedt’s catalyst and the weakest Si-C bond (78 kcal·mol^−1^) [39], it is prone to the scission of Si-CH_3_ in the first degradation stage. It is reasonable to speculate that a radical mechanism plays a role in the depolymerization of neat SR and SR/vinyl-GO nanocomposites. The free radicals generated during depolymerization are adsorbed and shielded by vinyl-GO due to its large specific area and two-dimensional geometry. These adsorbing and shielding effects could account for the improved *T_max,1_* with the increasing vinyl-GO content, as depicted in Table 1. The restricted cyclic transition state and the radical mechanism are consistent with the previously reported results of the organic peroxide-vulcanized cross-linked PDMS [40]. The scission of Si-CH_3_ catalyzed by the platinum compound occurs during its early depolymerization stage and subsequently forms a methylene bridge structure. 

The Si-O backbone might degrade via the cyclic oligomers at a higher temperature due to the restriction of the cyclic transition state exerted by the cross-linked structures and Karstedt’s catalyst. Furthermore, vinyl-GO is another critical factor influencing the formation of cyclic oligomers. Its interface and interfacial interaction with SR hinder the formation of a cyclic transition state, and the cyclic oligomers form at an evaluated temperature. This interfacial hindrance could account for the improved *T_max,2_* with the increasing vinyl-GO content.

### 3.4. Thermal Conductivity of SR/GO Nanocomposites

**Figure 6:**
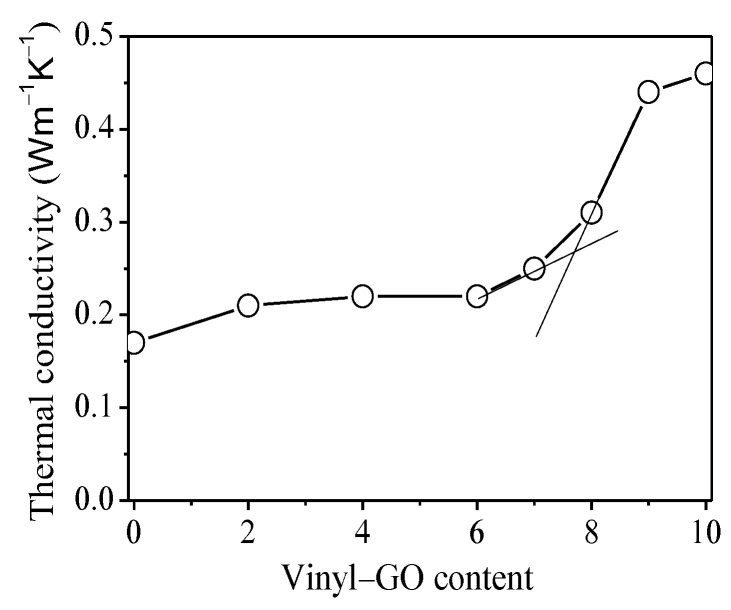
The relation of the thermal conductivity of SR/vinyl-GO nanocomposites with the vinyl-GO content.

It is generally accepted that an interconnected network plays a key role in thermal conductivity for thermally conductive nanocomposites [23,25,27,28,29]. The relation of the thermal conductivity of the nanocomposites with the vinyl-GO content is shown in Figure 6. When the vinyl-GO content is lower than six parts, the thermal conductivity of the composites remains almost invariable with the vinyl-GO content. This result suggests that the distribution of vinyl-GO in the SR matrix is isolated and random. As a result, the thermal conductive pathway is discontinuous in the nanocomposites with a lower vinyl-GO content. For the nanocomposites with over seven parts of vinyl-GO content, the thermal conductivity exhibits an obvious increase and continues to increase with the increasing vinyl-GO content. It can be inferred that vinyl-GO begins to interconnect and forms a continuous conductive pathway when its content is over seven parts. The thermal percolation threshold at 7.7 parts of vinyl-GO was obtained from the tangent intersection before and after the inflection point of the curve. The interconnected structure of vinyl-GO in the nanocomposites gradually becomes perfect over 7.7 parts, thereby yielding an uninterrupted thermal conductive pathway under the external loading.

### 3.5. Antistatic Property of SR/Vinyl-GO Nanocomposites

The material with a greater than 10^12^ Ω of resistance value is an electrical insulator, which is prone to generating accumulated charges on the surface and cannot be discharged. For the antistatic material with a varied resistance value from 10^6^ to 10^11^ Ω, the accumulated charges on its surface are not only less than that of the insulator but the charges that are generated are also relatively easy to release [41,42]. SR is identified as an electrical insulator because of the resistance value of 10^15^ Ω. When the vinyl-GO content is 2, 4, and 6 parts, the nanocomposites are electrical insulators due to their resistance value of 10^14^ Ω. For the nanocomposites with 7 parts and 8 parts of vinyl-GO, as shown in Table 2, they have resistance values of 10^12^ Ω, still suggesting electrical insulation. However, their resistance value of 10^12^ Ω indicates the interconnection of vinyl-GO, which is consistent with the discussion of the above thermal conductivity. An interconnected network formed by nano fillers plays a key role in efficient electron transport for antistatic nanocomposites. An interconnected structure meeting the thermal conductive pathways is still appropriate for electron transport. Relative to the eight parts of vinyl-GO content in the composites, the resistance value declines four orders of magnitude for nine parts of vinyl-GO, suggesting the formation of a preferable electron transport pathway. The electron transport prefers the interconnected vinyl-GO network, avoiding the SR matrix because of its higher electrical resistivity. Consequently, the nanocomposites exhibit an efficient antistatic property when the vinyl-GO content reaches nine parts or more.

### 3.6. Morphological Characterization of SR/GO Nanocomposites

**Figure 7:**
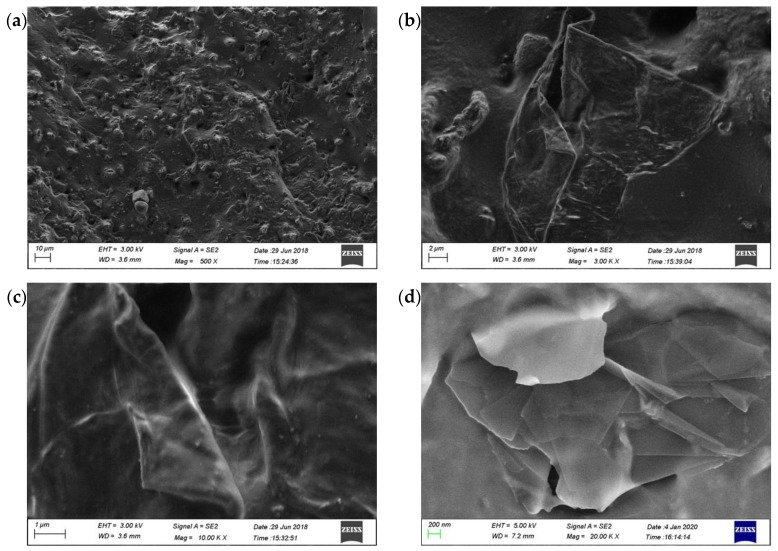
SEM images of SR/vinyl-GO nanocomposites with varied vinyl-GO contents. (**a**–**c**), 8 parts of vinyl-GO content. (**d**), 10 parts of vinyl-GO content.

To estimate the dispersion of vinyl-GO in the SR matrix, the micromorphology of fresh tensile fracture surfaces of SR/vinyl-GO nanocomposites was observed using SEM. As can be seen from Figure 7a, for the nanocomposites with eight parts of vinyl-GO, the interface of SR with vinyl-GO is not clear, and a uniform dispersion of vinyl-GO in the SR matrix is also observed. In addition, the monolayer of vinyl-GO observed in the nanocomposites (Figure 7b,c) exhibits a wrinkled, twisted, and folded surface, increasing its interfacial area with SR and subsequently yielding a considerable interfacial interaction that assists its dispersion in the SR matrix. Based on these results, it is reasonable to speculate that vinyl-GO is firmly embedded and has improved compatibility with the SR matrix. Additionally, the cross-linking degree of SR might suit the character of vinyl-GO and allow for the existence of its monolayer. However, when the vinyl-GO content is 10 parts, the aggregation of vinyl-GO in the SR matrix occurs, as shown in Figure 7d. Based on the above results of the thermal conductivity and antistatic properties, it is rational to deduce that the vinyl-GO content must be over eight parts to satisfy the thermally conductive and antistatic properties of the nanocomposites.

## 4. Conclusions

Vinyl-GO with a favorable hydrophobic surface plays a significant role in reinforcing the properties of SR/vinyl-GO nanocomposites. Within the tested contents, the nanocomposites showed a linear growth of the tensile properties with the increasing vinyl-GO content and exhibited a much higher thermal stability than neat SR. A threshold value of eight to nine parts of vinyl-GO in the nanocomposites exists, wherein the thermal conductivity and antistatic properties of the nanocomposites showed an abrupt increase. The addition of 9 parts of vinyl-GO increased the thermal conductivity to 0.44 from 0.17 W/m^−1^·K^−1^ of neat SR and the surface resistance value to 10^8^ from 10^14^ Ω of neat SR. The SEM image of the nanocomposites with eight parts of vinyl-GO suggests a uniform dispersion of vinyl-GO in SR matrix. However, when the vinyl-GO content was 10 parts, multilayer aggregation of vinyl-GO in the matrix occurred. Eight to nine parts of vinyl-GO is a preferable content suitable for the thermal conductivity and electron transport of the nanocomposite.

## Figures and Tables

**Table 1:** TGA data of neat SR and SR/vinyl-GO nanocomposites.

Vinyl-GO Content	*T_max,1_*^1^/°C	*T_max,2_*^1^/°C	Residual Ratio/%
0	381.6	507.8	23.5
2	435.5	613.9	35.2
6	430.8	580.1, 632.6	45.1
8	440.6	635.2	56.1
10	506.1	708.0	57.3

^1^ defined as the temperature of the maximum weight loss rate.

**Table 2:** The surface resistance values of SR/vinyl-GO nanocomposites.

Vinyl-GO Content	Surface Resistance Value/Ω
0	10^15^
2	10^14^
4	10^14^
6	10^14^
7	10^12^
8	10^12^
9	10^8^
10	10^7^

## Data Availability

Not applicable.
